# Diversity of *Bartonella* and *Rickettsia* spp. in Bats and Their Blood-Feeding Ectoparasites from South Africa and Swaziland

**DOI:** 10.1371/journal.pone.0152077

**Published:** 2016-03-21

**Authors:** Muriel Dietrich, Mabotse A. Tjale, Jacqueline Weyer, Teresa Kearney, Ernest C. J. Seamark, Louis H. Nel, Ara Monadjem, Wanda Markotter

**Affiliations:** 1 Department of Microbiology and Plant Pathology, University of Pretoria, Pretoria, South Africa; 2 National Institute for Communicable Disease, National Health Laboratory Services, Johannesburg, South Africa; 3 University of Pretoria, Pretoria, South Africa; 4 Ditsong National Museum of Natural History, Pretoria, South Africa; 5 University of the Witwatersrand, Johannesburg, South Africa; 6 AfricanBats, Kloofsig, South Africa; 7 Centre for Wildlife Management, University of Pretoria, Pretoria, South Africa; 8 University of Swaziland, Kwaluseni, Swaziland; University of Western Ontario, CANADA

## Abstract

In addition to several emerging viruses, bats have been reported to host multiple bacteria but their zoonotic threats remain poorly understood, especially in Africa where the diversity of bats is important. Here, we investigated the presence and diversity of *Bartonella* and *Rickettsia* spp. in bats and their ectoparasites (Diptera and Siphonaptera) collected across South Africa and Swaziland. We collected 384 blood samples and 14 ectoparasites across 29 different bat species and found positive samples in four insectivorous and two frugivorous bat species, as well as their Nycteribiidae flies. Phylogenetic analyses revealed diverse *Bartonella* genotypes and one main group of *Rickettsia*, distinct from those previously reported in bats and their ectoparasites, and for some closely related to human pathogens. Our results suggest a differential pattern of host specificity depending on bat species. *Bartonella* spp. identified in bat flies and blood were identical supporting that bat flies may serve as vectors. Our results represent the first report of bat-borne *Bartonella* and *Rickettsia* spp. in these countries and highlight the potential role of bats as reservoirs of human bacterial pathogens.

## Introduction

In recent years, the emergence of bat-borne pathogens has focused interest and stimulated further research. Several studies have shown pathogen spillover from bats to humans, leading in some instances to significant morbidity and mortality, as demonstrated for example with Marburg, Nipah and Hendra viruses [1]. In addition to viruses, bats have been reported to carry vector-borne bacteria, such as *Bartonella* and *Rickettsia* spp. [2], as well as ectoparasites capable of feeding on humans, however, their zoonotic threats remain poorly understood.

*Bartonella* and *Rickettsia* spp. are intracellular bacteria that are associated with a growing spectrum of emerging diseases in humans, such as life-threatening endocarditis and spotted and typhus fevers [3,4]. *Bartonella* species are mainly transmitted to humans through faeces of blood-feeding arthropods, such as fleas, ticks and lice, after superficial scratching of their skin, or directly by the bite of these ectoparasites. These bacteria infect a large range of mammals and are increasingly reported in bats and their ectoparasites [5–10]. Recently, the human pathogen *Bartonella mayotimonensis* has been associated with bats in the Northern Hemisphere [11]. For *Rickettsia* spp., only serological evidence of infection in bats has been reported [12] as well as the detection of *Rickettsia* DNA in bat ticks and flies [6,13,14]. Several bat ectoparasites, including ticks (Acari) and flies (Diptera), have been found to carry *Bartonella* and *Rickettsia* spp., some of them being identical to the genotypes found in bats, suggesting a vector-borne transmission. Despite these findings, the distribution and diversity of bat-borne *Bartonella* and *Rickettsia* spp., as well as the role of bats in transmission to humans remains poorly understood, especially in Africa where the abundance and diversity of bats is high [15].

To better understand the role of bats and their ectoparasites as reservoirs of bacterial pathogens, we investigated *Bartonella* and *Rickettsia* spp. presence using molecular detection tools in a large range of bat species across South Africa and Swaziland. We further assessed the genetic diversity of bat- and ectoparasite-borne *Bartonella* and *Rickettsia* spp. and their relatedness compared to others mammalian strains.

## Materials and Methods

### Bat sampling

**Fig 1:**
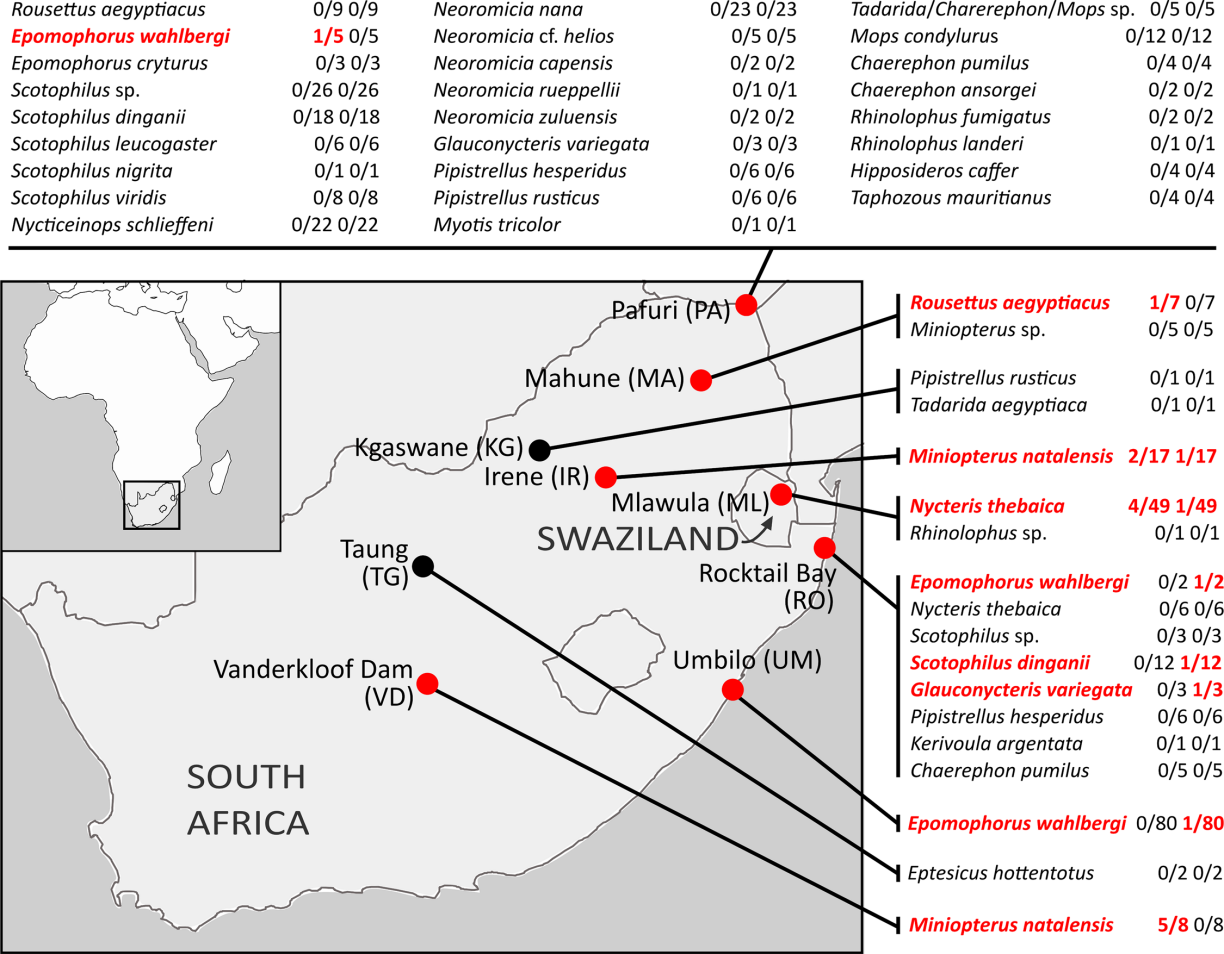
Detection of *Bartonella* and *Rickettsia* spp. in blood samples of bats from South Africa and Swaziland. Sampling location abbreviations are indicated in parentheses. The number of positive/tested samples is indicated for *Bartonella* and *Rickettsia* spp. respectively. Bat species that tested positive are highlighted in bold. Pafuri is located in the northern region of the Kruger National Park and ‘Kgaswane’ refers to the Kgaswane Mountain Nature Reserve. The map was drawn using the freeware PanMap software: http://www.pangaea.de/Software/PanMap).

From 2007 to 2012, bat sampling was conducted at nine different locations across South Africa and Swaziland at bat caves and open vegetation (Fig 1 and S1 Table). Bats were trapped during emergence at dusk using harp-traps and or mist nets and morphologically identified using taxonomic keys [15,16]. Bats were anesthetized using ketamine injection. Blood was sampled using cardiac puncture for both fruit and small insectivorous bats. Whilst individual bats were being anaesthetized, the body and fur of each bat was scanned for visible ectoparasites, which were subsequently collected in dry tubes and immediately stored in liquid nitrogen. When necessary, bats were kept in the holding bags for allowing a recovery period from anesthesia (< 30 minutes) and were then released at the site of capture by allowing them to fly freely. For some individuals, bats were euthanatized by a ketamine overdose and taken as voucher specimens that were lodged in museum collections (see details in S1 Table). Blood samples were collected in serum separator tubes (MiniCollect® Microtubes, Greiner Bio-One) and centrifuged at 8000 g for 5 mins to separate the red blood cells from the serum. Samples were stored in liquid nitrogen in the field and then transferred to -80°C freezers in the lab.

### Ethics statement

The sampling protocol was approved by the University of Pretoria Animal Ethics committee (EC054-14) following guidelines of the South African National Standard (SANS 10386:2008). Catching and collecting was carried out in strict accordance with the terms of research permits issued by national authorities: 000039 NW-07 for Taung, Kgaswane (North West, South Africa) Department of Agriculture, Conservation, Environment and Tourism, North West province, RB/2010/04 for Pafuri (Kruger National Park, Limpopo, South Africa) South African National Parks, OP 500/2010 for Rocktail Bay (St. Lucia, KwaZulu-Natal, South Africa) Ismangaliso Wetland park authority and Ezemvelo KZN wildlife, CPB6-003767 for Mahune (Limpopo, South Africa) issued by the Department of Economic Development, Environment & Tourism, and from the Swaziland National Trust Commission for Mlawula Game Reserve, Swaziland.

### Laboratory procedures

Genomic DNA was extracted from 100 μl anticoagulated blood, or whole ectoparasite specimens cut into small pieces, using the Qiagen DNeasy blood and tissue kit (Qiagen, Germany) according to the manufacturer’s instructions. The presence of *Bartonella* spp. was detected using a conventional PCR targeting the partial citrate synthase (*gltA*) gene [17]. For *Rickettsia* spp., we used a real-time PCR also targeting the *gltA* gene [18]. Positive *Bartonella* samples were sequenced using the same primers. Positive *Rickettsia* samples were further subjected to a nested PCR targeting the partial *gltA* gene using specific primers PanRick2-for and RpCS1258 [18], and NgltF (5’-GTATATTCCTAAATATATAGC-3’) and NgltR (5-GTTCTATTGCTATTTGTAAG-3’) (this study). All PCRs were run with a negative (water) and positive (i.e. plasmids containing the targeted gene fragment of *Bartonella henselae* and *Rickettsia conorii* respectively) controls. Molecular identification of ectoparasites was performed using the amplification of the cytochrome oxidase I (COI) fragment [19] and a BLAST search. Sequencing of the amplicons was performed on an Applied Biosystems 3500xl (Life Technologies, Carlsbad, US) at the DNA Sequencing Facility of the Faculty of Natural and Agricultural Sciences (University of Pretoria).

### Phylogenetic analyses

We used the freeware CLC Sequence Viewer 6 to align the *Bartonella* sequences generated in this study with 132 reference sequences representing the major *Bartonella* species and samples from bats worldwide. For *Rickettsia* spp., we used all the positive samples as well as 33 reference sequences representing the major *Rickettsia* species, including sequences from one bat tick [6] and bat flies [14]. JMODELTEST 2.1.4 was used to search for the best-fit model of nucleotide substitution for both *Bartonella* and *Rickettsia* alignments, using the Akaike Information Criterion (AIC) [20]. Phylogenetic trees were constructed based on the maximum-likelihood (ML) method, with 1000 bootstraps, using PHYML 3.0 [21].

## Results

### Bat samples and bacterial DNA detection

In total, 384 blood samples were collected from 29 bat species representing eight families, including both insectivorous and frugivorous bat species (Fig 1). Additionally, 14 blood-feeding ectoparasites, collected from different individuals of the frugivorous bat *Rousettus aegyptiacus* in Mahune, were analyzed. Ectoparasites collected from insectivorous bats were not tested. Overall, 13 blood samples were positive for *Bartonella* spp. and six for *Rickettsia* spp. (Fig 1). Four bat species were positive for *Bartonella* spp.: *Miniopterus natalensis*, *Nycteris thebaica*, *Epomophorus wahlbergi* and *Rousettus aegyptiacus*, and five for *Rickettsia* spp.: *M*. *natalensis*, *N*. *thebaica*, *E*. *wahlbergi*, *Scotophilus dinganii* and *Glauconycteris variegata*. We found one individual (*N*. *thebaica* from Mlawula, Swaziland) co-infected with both bacteria. Ectoparasites collected from *R*. *aegyptiacus* were identified as Nycteribiidae flies (99% similarity with *Eucampsipoda* spp.; Genbank accession numbers: KR997992-KR998001) and fleas (87% similarity with *Odontopsyllus* spp.; KR997991); five flies and one non-identified specimen (AA028) were *Bartonella*-positive and all were *Rickettsia*-negative. The flea sample was negative for both *Bartonella* and *Rickettsia* spp.

### Genetic diversity of *Bartonella* and *Rickettsia* spp.

**Fig 2:**
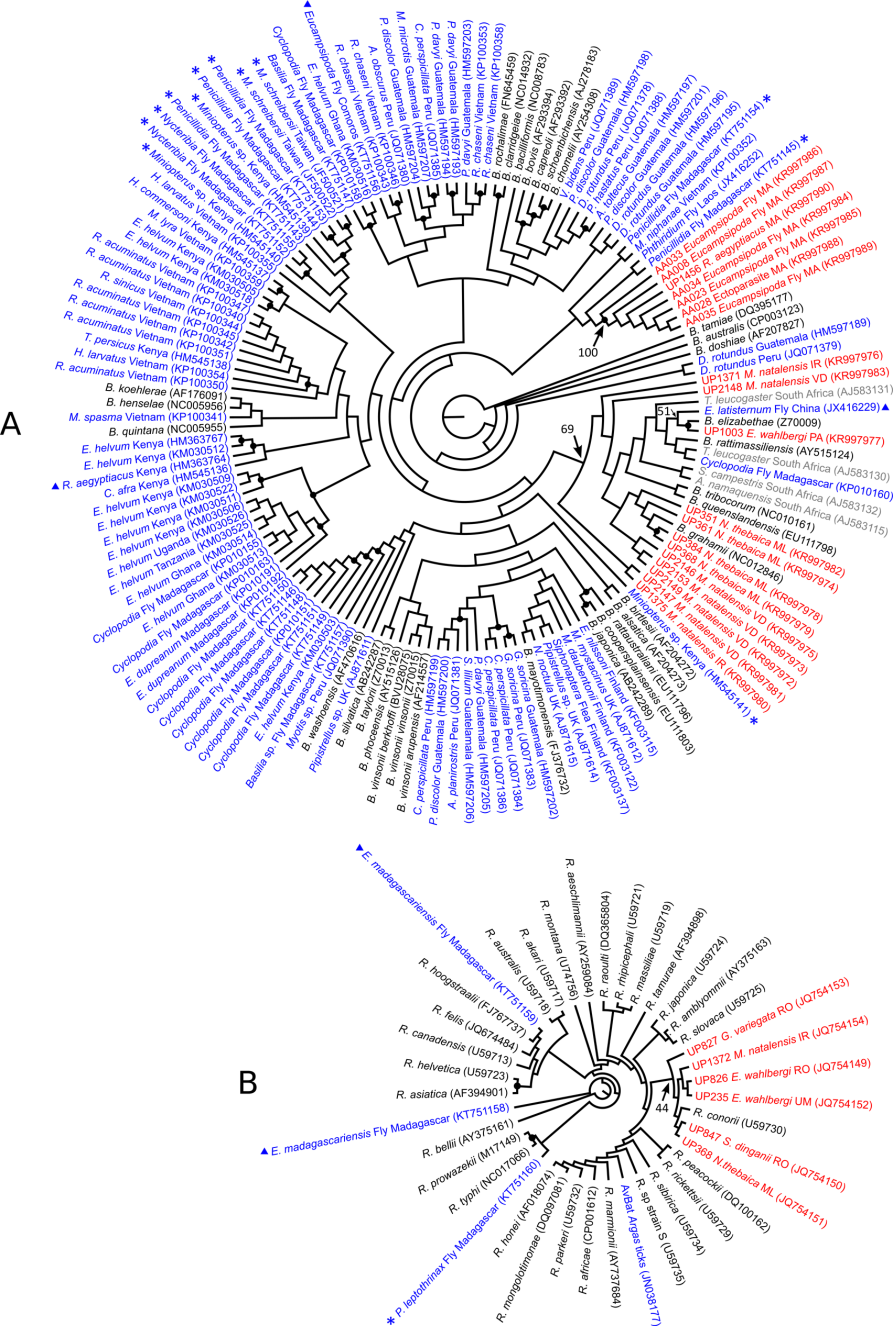
**Phylogenetic relationships of Bartonella (A) and Rickettsia (B) spp. detected in bats and their ectoparasites from South Africa and Swaziland.** Black dots indicate bootstrap > 0.75. Bootstrap values for nodes of interest are indicated by an arrow. Trees were built under the TIM3+G and TIM1+G models of evolution, for *Bartonella* and *Rickettsia* spp. respectively. The sequences generated in this study are in red and are coded with the sample ID, the host species and geographic location abbreviation as indicated in Fig 1. Reference sequences (retrieved from GenBank) corresponding to bat- and rodent-associated samples are in blue and grey, respectively. Sequences associated with *Miniopterus* and *Rousettus* bats are denoted by an asterisk (*) and a triangle (▲) respectively. GenBank accession numbers are indicated in parentheses.

After trimming of the sequences for quality and alignment with references, phylogenetic analyses were conducted on a 308-bp and 202-bp fragment of the *gltA* gene, for *Bartonella* and *Rickettsia* spp. respectively. Fig 2A revealed two main genetic groups among our *Bartonella* samples. The first group included samples from *N*. *thebaica* and *M*. *natalensis* (from different localities), which were closely related to *B*. *grahamii* (mean sequence similarity to *B*. *grahamii* = 98.2%) but distinct from *Bartonella* spp. previously reported in other *Miniopterus* bats and their flies from Kenya, Madagascar and Taiwan. The first group also included a *Bartonella* genotype detected in *E*. *wahlbergi* which grouped with *B*. *elizabethae* (100% sequence similarity on the 308bp analyzed) and a sequence from an *Eucampsipoda* fly collected from *Rousettus leschenaultii* in China, although the bootstrap value at the node was low (i.e. 51%). Moreover, this *Bartonella* genotype from *E*. *wahlbergi* was quite different from *Bartonella* spp. found in South African rodents [22] (mean sequence similarity = 94.9%). The second group showed that *Rousettus aegyptiacus* and their flies were infected with a similar *Bartonella* spp., but this well-supported genetic cluster did not group with any known *Bartonella* species and was distinct from those previously detected in the same bat species in Kenya [17], or from *Rousettus obliviosus* flies (*Eucampsipoda theodori*) in the Union of Comoros [14].

Analysis of the *gltA* gene fragment in *Rickettsia* spp. only allowed the identification at the group level and showed that our samples can be classified as spotted fever group *Rickettsia* spp. (Fig 2B). Despite low bootstrap supports, our samples clustered together and with *R*. *conorii* (mean sequence similarity of 99.5% between our samples and this species). Moreover, our samples were distinct from the bat-associated *Rickettsia* spp. described from the tick *Argas vespertilionis* in France [6], and from flies collected on *Rousettus* and *Miniopterus* bats in Madagascar [14].

## Discussion

We provide evidence of *Bartonella* and *Rickettsia* spp. infections in different frugivorous and insectivorous bat species and their blood-feeding ectoparasites from South Africa and Swaziland. Our results represent the first report in these countries and highlight the potential role of bats as reservoirs of human bacterial pathogens [11].

*Bartonella* genotypes identified in this study were distantly related to those previously described from bats, especially in other African countries, such as Kenya or Madagascar [7,14,17]. Some of them were closely related to human pathogens such as *B*. *elizabethae* and *B*. *grahamii*, which are however associated to only a few reports of endocarditis and neuroretinitis or bilateral retinal artery branch occlusions, respectively [23–26]. These two *Bartonella* species are typically found in rodent hosts, suggesting potential transfer between rodents and bats. However, according to our phylogenetic analyses, the respective genotypes infecting South African rodents [22] and bats seems to be different.

Moreover, different levels of host specificity seem to occur. Active interspecies transmission of *Bartonella* species within shared roosts of insectivorous bat species may contribute to a lack of host-specificity, as seen in the similarity of the *Bartonella* spp. found in *M*. *natalensis* and *N*. *thebaica*, and as previously suggested for bats in Guatemala and Vietnam [5,27]. In contrast, *Bartonella* spp. from *R*. *aegyptiacus* bats were distinct and unique from all other *Bartonella* species. The same result was previously shown for *R*. *aegyptiacus* in Kenya [17], but with a completely distinct genotype to that of those from South Africa. As our analysis was based on a single gene fragment with relative short length, further attempt to isolate the bacteria and obtain a better genetic characterization will be needed to confirm these results.

Available data on *Rickettsia* spp. infections in bats is based on serologic surveys [12] or detection of DNA in ticks and flies [6,13,14]. For example, a study in Brazil reported a significant degree of seroconversion against different *Rickettsia* spp. antigens in bats [12]. Additionally, infection with spotted fever group *Rickettsia* spp. have been reported in *Carios kelleyii* and *Argas vespertilionis*, two tick species that are well adapted to urban areas and can feed on humans in North America and Europe, respectively [6,13]. Our study shows that *Rickettsia* spp. infections can also be detected in bat blood. Analysis of the partial *gltA* sequence data led to identification of bat-associated *Rickettsia* spp. closely related to *R*. *conorii*, and distinct from those previously identified in bat ectoparasites from Europe and Madagascar [6,14]. However, our analysis could not provide further genetic identification. Additional analyses are thus required to better describe bat-borne *Rickettsia* spp. and their potential relationship to human pathogens.

Generally, ectoparasite infestation is known as a common mode of *Bartonella* and *Rickettsia* spp. transmission. In Africa, the first report of *Bartonella* spp. in bat flies was from *Cyclopodia greefi greefi* collected from the straw-coloured fruit bat *Eidelon helvum* in Ghana [8]. Brook et al. [7] also reported *Bartonella* spp. in both bat blood and flies (*Cyclopodia dubia*) collected in *Eidolon dupreanum* from Madagascar. More recently, a metagenomic study conducted in Madagascar showed that *Bartonella* and *Rickettsia* spp. are main components of the microbiota of different bat fly species [14]. Here, we identified similar *Bartonella* spp. in *R*. *aegyptiacus* blood and *Eucampsipoda* flies, showing that these ectoparasites, at least, may probably serve as vectors of *Bartonella* spp. [28]. As our study is based on a non-representative sampling of bat ectoparasites, future studies should increase the number of tested samples and involve investigations in other parasites, such as ticks, in order to better identify the vector range of *Bartonella* and *Rickettsia* spp.

In southern Africa, *Bartonella* and *Rickettsia* spp. infections have been reported in humans and animals [29,30]. For instance, a recent study in South Africa, showed high *Bartonella* prevalences (i.e. 9.5% and 22.5%) in blood of healthy and HIV-positive human subjects, respectively [30]. Here, we found relative low infection rates in bat blood for *Bartonella* (3.3%) and *Rickettsia* (1.5%) spp., but 36% of bat flies were PCR-positive for *Bartonella* spp., although these results need to be confirmed with larger sample size. Bat ectoparasites are generally known to be highly host-specific which may limit the probability of feeding on other hosts [31,32], and thus suggests that the global risk of spillover of bat-borne *Rickettsia* and *Bartonella* spp. to other animal species, including humans, may be low. However, as a result of greater human activity in caves in Africa, for religious activities especially [33], there is an increased likelihood of closer contact between certain bat species and humans, and between bats and other animals. Detection of *Bartonella* and *Rickettsia* spp. in bat species and their ectoparasites highlights the need to study whether these bat-associated bacteria are responsible for the etiology of local undiagnosed illnesses in humans.

## Supporting Information

S1 Table Details of the blood samples analyzed for *Bartonella* and *Rickettsia* in South Africa and Swaziland. Not all individuals sampled were taken as museum vouchers, and some vouchers have not yet been lodged in a public repository; in such instances identification based on morphology was limited to external features only, and hence identification to species level was not always possible. Acronyms used in Sample ID and Field/Museum number: UP—Virological Research Group, University of Pretoria; ECJS—Ernest Seamark, AfricanBats; NC—Northern Cape, Davis Jacobs, University of Cape Town; TM- Ditsong National Museum of Natural History, Pretoria (formerly Transvaal Museum); CHIR KNP: Skukuza Biological Reference Collection.
(XLSX)
